# Increasing Productivity and Recovering Nutritional, Organoleptic, and Nutraceutical Qualities of Major Vegetable Crops for Better Dietetics

**DOI:** 10.3390/foods14020254

**Published:** 2025-01-15

**Authors:** Raju Lal Bhardwaj, Latika Vyas, Mahendra Prakash Verma, Suresh Chand Meena, Anirudha Chattopadhyay, Neeraj Kumar Meena, Dan Singh Jakhar, Sita Ram Kumawat

**Affiliations:** 1 College of Agriculture, Agriculture University, Sumerpur-Pali 306902, Jodhpur, India; 2 Directorate of Extension Education, Maharana Pratap University of Agriculture and Technology, Udaipur 313001, Rajasthan, India; 3 College of Agriculture, Sumerpur 306902, Rajasthan, India; 4 Pulses Research Station, Sardarkrushinagar Dantiwada Agricultural University, Sardarkrushinagar 385506, Gujarat, India; 5 College of Agriculture, SKN Agriculture University, Jobner 303329, Rajasthan, India; 6 Agricultural Research Station, Agriculture University, Mandor 342304, Jodhpur, India; 7 Agricultural Research Sub-Station, Agriculture University, Sumerpur-Pali 306902, Jodhpur, India

**Keywords:** chemical fertilizer, microorganisms, organic sources, plant growth-promoting microorganisms, soil biodiversity

## Abstract

The intensive use of chemical fertilizers for vegetable cultivation to achieve higher productivity causes soil degradation, resulting in an alarming decline (25–50%) in nutritional quality and a reduction in a wide variety of nutritionally essential minerals and nutraceutical compounds in high-yielding vegetable crops over the last few decades. To restore the physio-chemical and biological qualities of soil as well as the nutritional and nutraceutical qualities of fresh produce, there is a growing desire to investigate the remedial impacts of organic sources of nutrition. This study specifically focused on the impact of six different ratios of chemical fertilizers and organic sources with microbial inoculation on vegetable productivity, nutrition quality, and soil health parameters. Results show that replacing chemical fertilizers with organic sources in the presence of a microbial consortium supports the proliferation of the microbial population in the soil rhizosphere and improves the nutritional status and physico-chemical quality of soil, which is the area around the roots of plants where maximum nutrient uptake occurs. This combination of factors significantly recovers overall soil quality, increasing crop productivity by 13.58 to 18.32 percent in tomato, brinjal, and okra. Experimental findings likewise indicate that an assortment of organic sources with a microbial consortium significantly recovers the abundance of beneficial microbes and earthworms in the rhizosphere, which leads to an improvement in nutritional, organoleptic, and nutraceutical quality, with higher antioxidant contents in all three vegetables grown in arid climate conditions.

## 1. Introduction

Global vegetable production mainly depends on the application of chemical fertilizers, which boost crop productivity. On the other hand, they adversely affect soil biodiversity, which is crucial for food security and sustainability [1]. The world food crisis has led to the intensification of the practice of applying chemical fertilizers to further improve or sustain crop productivity [2]. India is the second-largest producer of vegetables in the world and accounts for 15 percent of total production, with its average production of 17 mt being low in comparison to many other producing countries. At present, 204.83 million metric tonnes of vegetables are produced from an area of 11.35 million hectares, with India being the largest producer of okra among vegetables, with significant production of tomatoes and brinjal as well [3]. Vegetables are an imperative part of a balanced diet and play a vital role in human nutrition and health security, being a rich source of various minerals, vitamins, dietary fibers, and biochemical components with low calorific value. Worldwide, more than two billion people are suffering from micronutrient deficiency, especially in iron, iodine, vitamin A, folate, and zinc [4]. The reason behind this nutritional dilemma is that important commercial high-yielding vegetables such as tomato, chili, potato, cucurbits, and okra have lost their original nutritional density by up to 25–50 percent or more, including the loss of sodium (52%), iron (50%), copper (49%), and magnesium (10%) in different fruits and vegetables [5] with excessive pesticide residual contamination [6] during the last eighty years (1940 to 2019) due to environmental, genetic, and field soil degradation. The considerable nutrient depletion and diminished organoleptic quality of vegetables have been identified worldwide as disturbing the delicate balance of field soil, reducing soil life activities, altering physicochemical properties [7], and degrading soil quality and nutritional availability [8]. This is due to the serious alterations in traditional natural farming that began long ago and the adoption of chemical farming, which involves the application of imbalanced mineral nutrients through chemical fertilizers alone [6]. Therefore, identifying operative fertilization stratagems that mitigate the detrimental effects of excessive fertilizer application has become an imperative need, specifically for vegetable production.

The integration of different soil management approaches is an emerging need for nutrient-dense vegetable production and improved productivity. Different soil management approaches include well-adjusted fertilization and recovering soil biodiversity [9], microbial inoculation [10], increasing earthworm density [6], recycling field waste [6], and cohesive land management practices [11]. The return of well-decomposed, organic substances to the soil for precise soil reactions and the recycling of farm waste can lead to the regaining of soil microbial diversity and can support the fixation, solubilization, and supply of nutrients from the soil to plants, obtain better water-holding capacity, and have a significant impact on carbon sequestration, which could help mitigate the effects of nutrient dilution in different vegetable crops. Several researchers have reported that organically nurtured vegetables contain more protein, vitamin C, phosphorus, potassium, iron, magnesium, and calcium, as well as higher-quality secondary metabolites, nutraceutical compounds [12], phenolic compounds, polyphenols, carotenoids, and antioxidants [6,13]. Fruits and vegetables harvested from naturally grown forests or uncultivated areas are more nutritious, with good organoleptic qualities due to the presence of additional quantities of many phytochemicals. Particularly, a plant growth-promoting microorganism (PGPM) consortium of *Azotobacter chroococcum*, *Azotobacter vinelandii*, *Derxia* sp., *Bacillus megaterium*, *Bacillus lichenformis*, and *Bacillus subtilis* bacteria has been documented as an early root colonizer, which augments plant growth and development through diverse mechanisms, including the increased mobilization of insoluble nutrients and enhanced iron obtainability in the rhizosphere. The consortium also plays a key role in N-fixation and the production of phytohormones (auxins, gibberellins, zeatin, ethylene, abscisic acid) and antibiotics [6]; the mineralization and uptake of nutrients and the synthesis of vitamins and amino acids [14]; and the bioremediation of heavy metals in contaminated soils and the antagonism of potential plant pathogens through the production of antibiotics, siderophores, and hydrolytic enzymes [15], all of which can help in enhancing the nutritional density and organoleptic quality of food crops [16].

All high-yielding vegetables require more nutrition to uphold crop potential levels; therefore, trimming down the chemical sources of nutrients negatively affects crop productivity. On the other hand, increasing the productivity of vegetables is urgently required to fulfill the daily requirements of a balanced diet for the growing population. Because of the above facts, integrated nutrient management arises as a feasible approach to mitigate the adverse impacts engendered by the over-application of chemical fertilizers and to sustain productivity and soil superiority. This approach is not envisioned to supplant chemical fertilizers in a short time; comparatively, it advises decreasing their application through the simultaneous practices of nutrient-rich organic substances. The suggestion has confirmed that such collective application improves soil fertility more efficiently than the exclusive use of either chemical or organic fertilizers [17]. Previous studies have shown that the combined application of organic substances with bio-organic agents and chemical fertilizers can increase soil organic matter, improve crop root vitality and resistance, promote crop nutrient absorption and accumulation, enhance crop yield and fruit quality [18], remarkably improve the external and internal quality of fruits [19] and significantly increase soil availability of nitrogen and phosphorus [2]. Furthermore, the total soluble sugars (24%) and vitamin C (57%) increased in tomatoes, when the crop was grown with a 25% reduced application of chemical fertilizer plus Trichoderma-enriched bio-organic fertilizer, while nitrate accumulation was reduced up to 62 percent [17]. The hypothesis behind the long-term experimentation is to examine whether the excessive use of chemical fertilizers negatively impacts the field soil environment, whereas organic manure with PGPMs has been shown to improve soil quality and sustain both crop growth and nutritional superiority. The current literature provides limited insights into the outcomes of reducing chemical fertilizers and nutrients supplied by the application of organic material combination of soil microbial consortium (PGPM) on the yield and quality of tomato, chili, and okra. Therefore, this study determined the impact of a combined fertilization strategy on plant growth, fruit yield, and quality and also identifies the optimal fertilization ratio for maintaining soil fertility and production sustainability.

## 2. Materials and Methods

### 2.1. Experimental Site and Treatment Application

Field experiments have been conducted at the College of Agriculture, Sumerpur-Pali (Rajasthan), India to evaluate the impact of organic sources of nutrient supply with or without microbial inoculation over inorganic nutrition on soil quality, crop growth, productivity, nutritional and organoleptic evaluation of tomato, brinjal, and okra during two successive seasons from February to August 2023 and 2024. The center is located at 73°05′ east longitude and 25°09′ north latitude with an altitude of 272.0 m above Mean Sea Level (MSL). The location is grouped under an arid region with rainfall of 356.8 mm, RH 48.25 percent, with a maximum temperature of 33 °C and a minimum of 19.5 °C during experimentation. The experiment was laid out in a Randomized Block Design with six treatments and four replications. The treatments consisted of Field No. 1 (100% recommended dose of fertilizer (RDF) supplied by chemical source of nutrients); Field No. 2 (75% RDF by chemical source + 25% RDF by organic source); Field No. 3 (50% RDF by chemical source + 50% RDF by organic source); Field No. 4 (25% RDF by chemical source + 75% RDF by organic source); Field No. 5 (100% RDF by organic source); Field No. 6 (100% RDF by organic source + PGPM inoculation). The entire quantity of organic sources supplied by well-decomposed farmyard manure (FYM), vermicompost, and poultry manure (1:1:1), and a treatment-wise full dose of phosphorus, potash, sulfur, and 1/3 of nitrogen were applied during land preparation. The doses of nitrogen fertilizer as urea (46% N), phosphorus as Di-ammonium Phosphate (DAP) (48% P_2_O_5_ + 18% N), potassium as muriate of potash (60% K_2_O), and sulfur as sulfex gold (sulfur 80% *w*/*w*) were used. The rest of the nitrogen was applied in two equal splits as a top dress at 30 and 60 days after transplanting or seed sowing. PGPMs were collected from the plant microbiology laboratory, Department of Microbiology, IARI, New Delhi. Liquid cultures of the bacterial strains were prepared in nutrient broth at 28 °C. The concentration of bacterial cells was maintained at 109 CFU/mL. The culture was mixed with the recommended dose of vermicompost and applied before transplanting of tomato and brinjal, and seed sowing of okra. The plot size was 5 m × 90 cm on a raised bed with drip irrigation and adjacent plots and blocks were 1.0 m and 1.5 m apart, respectively. Data were collected from the middle rows of each plot, leaving one plant at both ends of the rows. All other recommended cultural practices including irrigation, gap filling, weeding, diseases, and pest management concerning tomato, brinjal, and okra production were performed as and when required.

### 2.2. Soil Quality Parameters

#### 2.2.1. Soil Sample and Nutritional Status

The nutritional status of soil was evaluated in the initiation of field homework (2019–2020) and the fourth year of the experimentation (2023–2024). The vertical soil of 0–30 cm (rhizosphere of vegetables) of soil layers was selected in each investigation field by the five-point sampling method, with four replicates. The soil samples were passed through a 100-mesh sieve after drying in the shade for chemical and nutritional propertys determination. The initial properties of the experimental field soil are presented in Table 1.

#### 2.2.2. Soil Physical Properties

The bulk density of soil (up to 30 cm depth) was determined by a core sampler with cores of 10 cm in height 5 cm in diameter and about 200 cm^3^ in volume [22]. Total porosity was determined in undisturbed water-saturated samples assuming no air was trapped in the pores. The soil moisture contents were determined gravimetrically at 105 °C for 24 h. The infiltration rate of soil in all treatments was measured by using a double-ring infiltrometer [27]. The water-holding capacity of soil was determined by the procedure of Gupta [28].

#### 2.2.3. Soil Chemical Properties

Soil pH and EC were measured by using a soil–distilled water suspension ratio of 1:2.5 (*v*/*v*) with a pH meter (NK-VI, SYSTRONICS^®^, Systronic India Pvt Ltd, Ahmedabad, India) and standard electrodes, respectively [23]. The soil organic carbon content was determined by the modified Walkley and Black rapid titration method [29]. The available N, P, and K content of the soil were analyzed as per the methods of Subbiah and Asija [25], Olsen et al. [26], and Jackson [23], respectively.

#### 2.2.4. Di-Ethylene-Tri-Amine-Penta-Acetic Acid (DTPA)-Extractable Micronutrient

The DTPA-extractable Fe, Mn, Zn, and Cu content of the soil was determined by the procedure of Lindsay and Norvell [30]. Ten grams of air-dried soil (˂2 mm) were weighed and transferred into a 125 mL polyethylene wide-mouth bottle. A total of 20 mL of DTPA extracting regent of air-dried soil (13.3 mL TEA, 1.967 g DTPA, and 1.47 g CaCl_2_.2H_2_O dissolved in 1 L and adjusted pH at 7.3) was added and shaken for 2 h in a rotary shaker (Thermo Fisher Scientific, Waltham, MA, USA). An aliquot was taken after filtering through Whatman 42 filter paper (Whatman International Ltd., Kent, UK) and estimated by atomic absorption spectrophotometer (SYS-813, Systronic India Pvt Ltd., Ahmedabad, India).

### 2.3. Soil Biological Population

For microbiological analysis, ten grams of field soil were diluted in a 10-fold series in sterile water. Using plate colony counts, viable bacterial populations were determined in NA agar containing 50 mg L^−1^ cycloheximide [31]. Using the serial dilution plate method [31], viable fungus propagules (CFU) and viable actinomycete propagules were determined in rose bengal agar containing 100 mg L^−1^ chloramphenicol [32] and in actinomycete isolation agar, respectively. A dilution blank series was first prepared, comprising sterile water for each series in 250 mL conical flasks containing 90 mL of sterile water and 8–10 test tubes containing 9 mL distilled water. One gram of soil sample was transferred in 9 mL sterile distilled water in a test tube (1:10) and then it was shaken properly. Thereafter, 1 ml of suspension was transferred from this tube to another tube containing 9 mL of sterile distilled water (1:100). Again, 1 mL of suspension was transferred from this tube to the tube containing 9 mL of sterile distilled water (1:1000). Using a fresh sterile micro-pipette, a 1 mL aliquot was prepared from the last dilution and poured into each of the three Petri dishes. Thereafter using the same pipette, aliquots prepared from the next two dilutions were transferred into Petri dishes for each dilution, then approximately 20 mL volume of molten media in each of the plates. After pouring, gently move the plates in a whirling motion to mix the contents, then allow the medium to solidify and incubate in a BOD incubator at 30° ± 2 °C in an inverted position. Nutrient agar plates for bacteria were incubated at 35–37 °C for 24 h and potato dextrose agar (PDA) plates for fungi were incubated at 25–27 °C for 48–72 h [33]. After the designated incubation period, the number of colonies per plate was enumerated in CFU/g using a digital colony counter. The standard procedures were followed in the viable count as recommended by Jagtap [33]. Total viable counts were determined employing the following formula for quantification. Total viable count = Average number of colonies × size of aliquot × dilution factor. The earthworm population (number of worms square meter^−1^) was observed by simple worm counting in distinctive areas up to 30 cm soil depth and the observation process was repeated up to four times.

### 2.4. Vegetative Growth and Yield Attributes

All the growth parameters were taken by scientifically described methods [34] from ten randomly selected competitive plants in each treatment and replication 15 days before the last harvest. The number of fruits per plant, fruit weight, and fruit yield per hectare were also recorded by using standard methods from ten randomly selected plants and averaged. The total income was calculated from yield multiplied by the average market rate during the period of fruit harvesting. Further, the net return was calculated by subtracting the cost of each treatment from total income. The benefit–cost ratio was calculated by dividing net income by the total cost of cultivation [34].

### 2.5. Proximate Composition and Carbohydrates

The moisture percentage of fresh fruits was calculated by the loss in weight of the sample divided by the weight of the fresh sample and expressed in percentage [35]. Ash content (%) in the samples was estimated by the standard method [36]. The total nitrogen (%) was estimated by a standard method of AOAC [36], using the Gerhardt Fully Automatic Nitrogen Estimation System. The crude protein was calculated by using the conversion factor of N × 6.25. The crude fiber in vegetable samples was estimated by utilizing the automatic Fibra Plus system (Pelican Equipments, Chennai, India) through pursuing the standard method of analysis [35]. Crude fat was estimated by the standard method [36] using the Automatic SOCS plus Solvent Extraction Apparatus. The total carbohydrate was calculated with the following formula:

$$\begin{array}{l} Total\;carbohydrates\;\left(\%\right)\\ \;\;\;\;\;\;\;\;\;\;\;\;\;\;\;\;\;\;\;\;\;\;\;\;=100\;-\;[moisture\;(\%)+crude\;protein\;(\%)+crude\;fat\;(\%)+crude\;fibre\;(\%)\\ \;\;\;\;\;\;\;\;\;\;\;\;\;\;\;\;\;\;\;\;\;\;\;\;+total\;ash\;(\%)] \end{array}$$

Total soluble sugars were determined by the method of Yemm and Willis [37], and reducing sugars by the Somogyi modified method [38]. The amount of non-reducing sugar was calculated as the difference between total sugars and reducing sugars. The starch from sugar-free pellets was estimated by the method of Clegg [39].

### 2.6. Nutritional Quality and Minerals

Total nitrogen (N) was determined by the micro-Kjeldahl method, whereas the total phosphorus was analyzed using a vandate-molybdate reagent, and absorbance was measured using a Microvolume Spectrophotometer [(COPTIZEN NanoQ), KLAB (Korea) Co. Ltd., Seoul, Republic of Korea] at 420 nm. The level of total potassium was determined using a flame photometer. The levels of iron (Fe), zinc (Zn), calcium (Ca), and magnesium (Mg) content in acid-digested samples were analyzed using an Atomic Absorption Spectrophotometer (SYS-813, Systronic India Pvt Ltd., Ahmedabad, India), following the procedure outlined by Lindsey and Norwell [40].

### 2.7. Organoleptic Evaluation of Fresh Fruits

Freshly harvested vegetables were subjected to organoleptic evaluation concerning color, appearance, aroma, texture, taste, and overall acceptability by a panel of 7 semi-trained judges, using the 9-point hedonic scale [41].

### 2.8. In Vitro Digestibility

The in vitro starch digestibility was assessed by Singh et al. [42] and protein digestibility was determined by the improved method of Akeson and Stahoman [43] and Singh and Jambunathan [44] with some modifications.

### 2.9. Nutraceutical Quality and Antioxidant Content

The spectrophotometer assay for the quantitative determination of total flavonoid content was carried out as described by Zhishen et al. [45]. The concentration of total phenolics in the methanolic extracts was determined by the Folin–Ciocalteau method [46]. DPPH free radical content was determined by the method followed by Brand-Williams et al. [47] as previously described by Tadhani and Subhash [48]. The Ferric Reducing Antioxidant Power (FRAP) Assay, originally developed by Benzie and Strain [49], was utilized to determine the total antioxidant capacity of the methanolic extracts. The ABTS radical scavenging activity was measured following a modified version of the method developed by Re et al. [50].

### 2.10. Statistical Analysis

Analysis of variance was performed for each attribute; means comparisons were carried out by mathematical calculation in the form of “percent change” and separated by a post hoc Tukey’s test and statistically significant differences were determined at the significance level of α = 0.05 (*p* < 0.05). To obtain a general comprehensive characterization of the nutrient supply sources, treatments for different traits were subjected to principal component analysis (PCA) based on correlations. The statistical treatments of the data were performed using the JMP software (SAS Institute Inc., Cary, NC, USA, version 8).

## 3. Results

### 3.1. Effects on Soil Nutritional Status

The nutritional quality of experimental field soils up to 0–30 cm depth, in terms of the available status of soil organic carbon, N, P, K, S, Zn, Fe, Cu, and Mn, was significantly improved with respect to the increase in the proportion of organic sources and incorporation of microbial consortium in place of chemical sources of nutrients (Table 2). The overall nutritional status of the experimental fields was ranked as Field No. 6 > Field No. 5 > Field No. 4 > Field No. 3 > Field No. 2 > Field No. 1. The content of soil organic carbon in the experimental field soil had a significant upward trend from 0.29% to 0.70% (141.38%) when crops were nourished by organic sources with microbial inoculation (Field no. 6), during the four years of experimentation. Similarly, the available nitrogen (2.27%), available phosphorus (93.33%), potassium (38.42%), zinc (237.14%), iron (82.16%), copper (86.87%), and manganese (41.57%) were increased significantly from the initial nutrient availability status in comparison to the nutrient supply by pure organic sources with microbial inoculation (Field No. 6) during the experimental period (2019–20 to 2023–24). A significant change was also reported by the treatment effects and 100% RDF supplied by organic sources with microbial inoculation, increasing the status of nutrient availability, such as phosphorus 26.5 to 72.5 Kg/ha (173.5%), potassium 206.5 to 312.0 Kg/ha (51.90%), zinc 0.23 to 1.18 ppm (413.04%), iron 1.36 to 3.37 ppm (147.79%), copper 0.46 to 1.12 ppm (143.48%), and manganese 6.40 to 8.48 ppm (32.50%). Conversely, the nitrogen content of 237.5 to 225.0 Kg/ha (−5.26%) and sulfur 8.62 to 7.11 Kg/ha (−17.52%) of the experimental field continually decreased with increasing organic sources of nutrition (Table 2).

### 3.2. Effects on Soil Physicochemical Quality

Statistical analysis of the data illustrated in Table 2 showed that the physicochemical qualities of the experimental field soil, such as EC, pH, bulk density, particle density, porosity, infiltration rate, water holding capacity, and soil moisture, were encouragingly influenced by different nutrient supply sources during the four years of the experiment.

The test soil was originally moderately alkaline with pH 8.4 and lower electrical conductivity (EC 0.13) during the commencement of the experiment. The soil pH faintly increased consistently (8.4 to 8.5) as a result of the nutrient supply by application of chemical fertilizers, whereas it significantly decreased (9.52%) with the increased application of organic manures with injection of the microbial consortium, and in Field No. 6, soil pH decreased from 8.40 to 7.60 during the four years of experimentation (Table 2). The EC value under the chemical source treatment was also at the lowest level (0.11 mS/cm), much lower than that of Field No. 2, Field No. 3, Field No. 4, Field No. 5, and Field No. 6 treatment by 36.36%, 72.73%, 100%, 190.91%, and 327.27%, respectively. The physical qualities of experimental fields were amended by the treatment of nutrient supply through organic sources with the incorporation of microbial consortium (PGPMs), which also helps to decrease soil bulk density (1.43 to 1.32) and particle density (2.39 to 2.27), whereas it considerable enhances soil porosity (7.04%), infiltration rate (108.31%), mean water holding capacity (47.88%), moisture content (33.84%) and allows outcomes such as saving more water (32.38%) during crop production in comparison to nutrient supply by the chemical sources (Field No. 1), because soil organic carbon is critical to improving soil physical structure for better crop production.

### 3.3. Effects on Soil Biological Population

Data recorded in Table 2 show that different nutrition supply sources had a substantial effect on the soil biological population in terms of fungi, bacteria, actinomycetes, and earthworms in the field soil. In this direction, the complete nutrition supplied by organic sources in combination with the PGPMs significantly increases in soil biological population than those of other combinations. It is also observed that the 100 percent and 75 percent RDF were supplied by chemical sources (Fields No. 1 and 2), and no biological population was reported during the fourth year of experimentation. This increases the biological population (fungi, bacteria, Trichoderma, actinomycete, and earthworm) with respective increases in organic sources of nutrition in Fields no. 4, 5, and 6 during experimentation. Most significantly, there was an increase in fungi (2966.7%), bacteria (1267%), Trichoderma (9600%), actinomycetes (1775%), and earthworms (49.15%) count when crops were nourished by 100% organic sources with the microbial consortium (Field No. 6).

### 3.4. Effects on Vegetative Growth and Yield Attributes

The vegetative growth and yield parameters were significantly affected by different nutrient supply sources in tomato, brinjal, and okra crops (Table 3). Significant and continuous declines in growth and yield attributes were reported with the replacement of chemical fertilizer by organic sources only, whereas the opposite results were reported when incorporating PGPMs with organic sources (FYM+ vermicompost+ poultry manure). There was a considerable increase in plant height (3.76%, 6.38%, 12.89%), number of leaves per plant (9.88%, 8.47%, 18.74%), root length (10.57%, 4.61%, 19.62%), number of roots per plant (27.31%, 43.79%, 54.88%), number of fruits per plant (13.75%, 10.04%, 16.95%), fruit weight (11.72%, 8.85%, 7.81%), fruit yield (18.32%, 13.58%, 16.23%), total income (16.02%, 13.52%, 16.02%) and net income (7.89%, 4.52%, 6.77%) when nutrients were supplied by organic sources with microbial inoculation (Field No. 6) for tomato, brinjal and okra fields, respectively. Due to the higher cost of cultivation of tomato, brinjal, and okra, the B: C ratio of Field No. 6 was reported to be lower (−23.89%, −23.86%, and −23.89%, respectively) in comparison to Field No. 1 (Table 3).

### 3.5. Effects on Nutritional and Organoleptic Quality

The proximate composition of vegetables presented in Table 4 showed that with the increase in the proportion of organic sources, the moisture content of fruits decreased non-significantly. In contrast, soluble solid content in the fruits showed an increased trend (Table 4). The highest content of crude protein (2.10%) in tomato, brinjal, and okra fruits was reported when crops were grown in Field No. 1, which was significantly (*p* < 0.05) higher than that of other treatments. Compared with chemical-based treatments, the crude fat, ash, and crude fiber content are augmented linearly with the increase in the proportion of organic sources. The highest increase of 44.12%, 35.38%, and 10.56%, respectively, was reported when nutrients were supplied by organic sources with the microbial consortium inoculation (Field No. 6). Total carbohydrate content in vegetables was marginally augmented with increased organic sources of nutrients supply. The highest total sugars (3.64%), reducing sugar (2.59%), and non-reducing sugars (1.05%) of the vegetables were reported in the crops grown in Field no. 6, at 227.93%, 338.98%, and 101.92%, respectively, higher than those of chemical source-supplied field produces. The average mineral contents increase in a range from 6% to 92.59% and can be ranked in the order of Field No. 6 > Field No. 5 > Field No. 4 > Field No. 3 > Field No. 2 > Field No. 1. The 100% organic sources of nutrient with microbial inoculation (Field No. 6) significantly recovered the mineral substances in tomatoes, brinjal, and okra, including increases in calcium content (6.0%), phosphorus (6.48%), iron (46.37%), zinc (92.59%), magnesium (15.17%), nitrogen (7.04%) and potassium (12.14%) in contrast to chemical sources of nutrient supply.

With the increase in organic source proportion, the organoleptic quality score of the fruits showed an upward trend and was significantly higher than the pure chemical sources of nutrient supply (Table 4). The organoleptic evaluation score of tomato, brinjal, and okra produced by 100% organic sources of nutrient supply with microbial inoculation treatment was the highest, which is attributed to the maximum score of appearance (7.81), color (8.37), aroma (7.91), taste (8.22), texture (8.22) and overall acceptability (8.30) compared with the control (Field No 1), representing an increase of 6.4%, 8.56%, 18.65%, 32.87%, 12.42% and 15.60%, respectively (Table 4). The in vitro protein digestibility was highest (78.04%) in 100% organic sources of nutrient supply with microbial inoculation treatment (Field No. 6), compared to the 100% chemical source of nutrient supply treatment (Field No. 1), with an increment of 4.78 percent. The in vitro starch digestibility of tomato, brinjal, and okra fruits in 100% organic sources of nutrient supply with microbial inoculation treatment was highest (70.99%), compared to the 100% chemical source of nutrient supply treatment, and was significantly (*p* < 0.05) increased by 11.34 percent.

### 3.6. Effects on Nutraceutical Quality and Antioxidant Content of Fruits

The nutrition supply source treatments significantly influence the substance of all phenolic acids, flavonoids, radicals scavenging, and antioxidant activities of vegetables (Table 5). Total flavonoid content in tomato fruits is significant and continues to increase when the crop is grown from Field No. 1 to Field No. 6 at about 37.56% and the content did not vary in Field No. 4 and Field No. 5. Total phenolic contents of fruits with different nutrient supply sources are significantly increased by about 41.14% and reached the highest value at 8.74 mg GAE/g fruit in Field No. 6, whereas the minimum value (3.15 mg GAE/g) is observed in the tomato fruits produce in Field No. 1. Further application of organic manure from the chemical field (Field No. 1) to 25% organic sources (Field No. 2) did not show significant variation but a significant increase was observed in Fields No. 3, 4, 5, and 6 compared to Field No. 1, and they also have a significant difference compared to each other.

When compared to the control, the radical scavenging activity (DPPH) of tomato fruits showed a slight and significant increase in Field No. 6 of about 38.27%, but the fruit produced from Field No. 2 (11.29 µmol Trolox/g) and Field No. 3 (11.49 µmol Trolox/g) had a non-significant difference compared to each other. The ferric-reducing antioxidant ability (FRAP) of tomato fruits also showed a similar increasing trend from Field No. 1 (14.73 µmol Trolox/g) to Field No. 6 (19.27 µmol Trolox/g) of about 30.82%. The ABTS value of the tomato fruits was pointedly affected, with an increase of 20.96% in crops produced in Field No. 6, in comparison to Field No. 1. Upon the succeeding increase in organic sources, the ABTS levels significantly increased, and the highest value (34.11 µmol Trolox/g) was measured in Field No. 6 tomato fruits, which was on par with Field No. 5 (33.66 µmol Trolox/g), showing an increase of about 20.96% and 19.36% compared to control (100% chemical sources). Similarly, brinjal crop was also grown in analogous fields, which have significant effects on the quantity of total flavonoids, phenolic content, radical scavenging activities, FRAP and ABTS values and considerably increased in Field No. 6, with 48.37% and 39.66%, respectively, in comparison to control (Field No. 1). Brinjal fruits which were produced in Field No. 6 have significantly higher nutraceutical and antioxidant quality than all other fields, but Field No. 5 closely followed, whereas the lowest value of all related parameters was recorded in Field No. 1 fruits. Nutrient supply sources affected the content of all phenolic acids, flavonoids, and antioxidant activities of okra fruits (Table 5). The highest total flavonoid content (3.62 mg QE/g) was reported in Field No. 6 okra fruits, which showed a significant increase in Field No 2 (1.61%), Field No. 3 (6.05%), Field No. 4 (13.31%), Field No. 5 (19.76%) and Field No. 6 (45.97%) as compared to Field No. 1. The total phenolic content of okra fruits also showed a notable variation, when supplying nutrients by several sources for crop production (Table 5). A minimum increase was observed when going from Field No. 1 to Field No. 2 (2.15%), whereas a huge change was reported when the crop was grown in Field No. 5 (30.82%) and Field No. 6 (48.75%) in comparison to Field No. 1 fruits. Radical scavenging activity (DPPH) of okra fruits also showed an increasing trend over increased organic sources of nutrients, reaching 49.17% more at Field No. 5 and 54.14% more at Field No. 6 (100% organic sources of nutrient + PGPMs inoculation). The ferric-reducing antioxidant ability (FRAP) of okra fruits showed the highest concentration at Field No. 6 (8.54 µmol Trolox/g) compared to Field No. 1 (6.15 µmol Trolox/g), with an increase of about 36.86%. The AA measured by ABTS showed a significant difference in supplying the nutrients by different levels of sources for okra crop production; it reached the highest and most significant value (24.85 µmol Trolox/g) measured at Field No. 6 okra fruits, which was closely followed by Field No. 5 (24.02 µmol Trolox/g) fruits, with an increase of about 26.33% and 22.12% compared to Field No. 1 (100% chemical sources) fruits.

### 3.7. PCA Biplot Analysis Links Nutrient Supply Sources with Soil Properties

**Figure 1:**
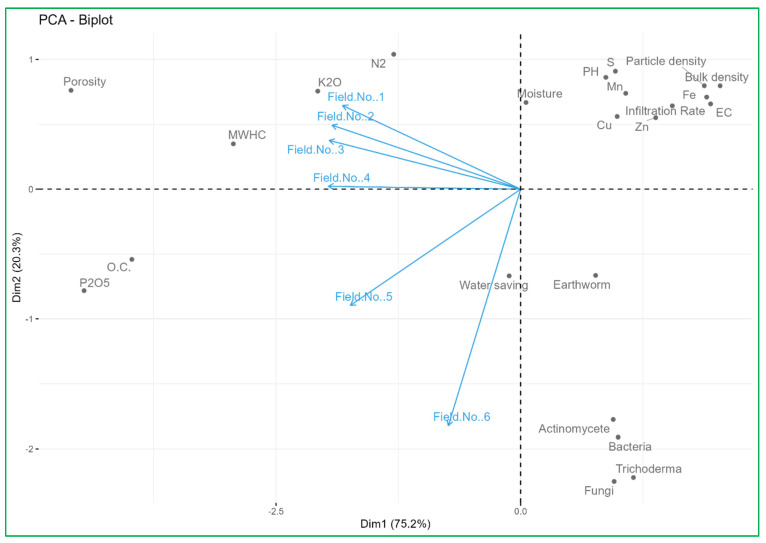
PCA biplot analysis links nutrient supply sources with soil properties.

To evaluate the associations between the effect of nutrient supply sources on field soil properties and the studied variables, a PCA biplot was performed. Principal component 1 (Dim 1) explained 75.2% of the total variance, while principal component 2 (Dim 2) was able to explain 20.3% of the total variance. Figure 1 shows the score and the loading plot, respectively. The PCA biplot provides a comprehensive visualization of the relationships between soil health parameters and the experimental field (Field No. 1 to Field No. 6), highlighting key patterns and variations. Field No. 1, Field No. 2, and Field No. 3 cluster closely, indicating similar soil characteristics, particularly concerning K_2_O and N_2_ concentrations, which align with moderate moisture levels and physical properties like mean water holding capacity and soil porosity. Field No. 4, Field No. 5, and especially Field No. 6 show more distinct profiles. Field No. 6, located further from the origin along the PC2 axis, is characterized by unique soil properties, possibly indicating issues like higher water-saving capacity, earthworm activity, and associations with beneficial microbial activity (actinomycetes, bacteria, fungi, Trichoderma), which suggest conditions closer to traditional organic fields. It also has all respective qualities that positively support the improvement of soil biodiversity and physical condition with higher fertility characteristics. The alignment of vectors such as organic carbon, earthworm count, and microbial activity (actinomycetes, bacteria, fungi) with Fields No. 5 and No. 6 indicates that these fields have higher organic matter and better soil health compared to the other fields. Conversely, parameters like pH, electrical conductivity (EC), and specific nutrient concentrations (Zn, Fe, Mn, Cu) show varying influence across the fields. Most significantly, including a positive correlation between the nutrient supply by organic sources and inoculation of different beneficial microbes in field soil resulted in a significant increase in soil organic carbon, and availability of essential nutrients (N, P, K, S, Zn, Fe, Cu and Mn) in the soil rhizosphere in Field No. 6. This biplot underscores the importance of tailored soil management practices: enhancing organic matter in fields with lower organic carbon, addressing compaction in Field No. 1, 2 and 3; optimizing nutrient management based on the unique profiles of each field. Implementing these targeted strategies can enhance soil health and sustainable productivity of the potential vegetable crops.

### 3.8. PCA Biplot Analysis Links Nutrient Supply Sources with the Nutritional and Organoleptic Quality of Vegetables

**Figure 2:**
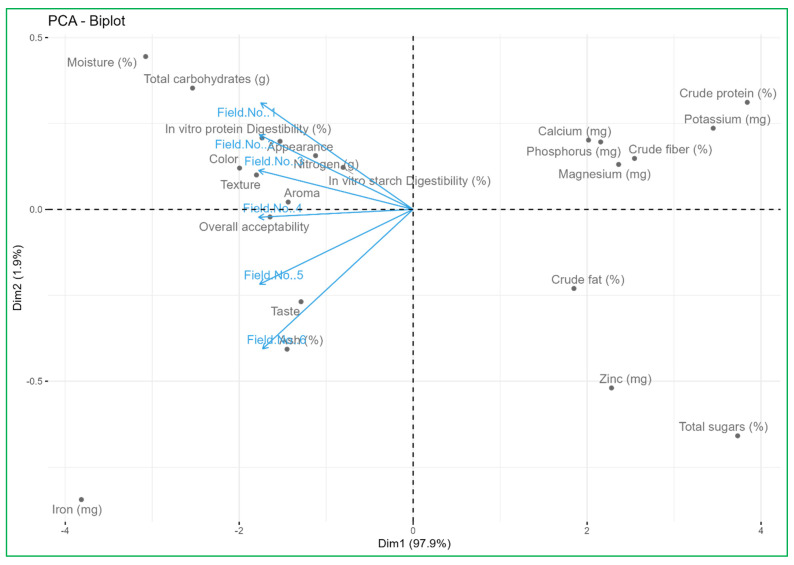
PCA biplot analysis links nutrient supply sources with the nutritional and organoleptic quality of vegetables.

To assess the relationships between the nutrient supply sources on the nutritional and organoleptic quality of vegetables and the studied variables, a PCA biplot was created. Principal component 1 (Dim 1) explained 100% of the total variance, while principal component 2 (Dim 2) was able to explain 0.0% of the total variance. Figure 2 shows the score and the loading plot, respectively. We conducted an integrative analysis of the significant positive correlations that existed between the soil rhizosphere nutrient indicators (availability of N, P, K, S, Zn, Fe, Cu, and Mn) and the content of crude fat, ash, crude fiber, sugars, iron, zinc, magnesium in vegetables with a significant increase in organoleptic and nutraceutical quality due to higher quantity of antioxidant contents. The PCA biplot presented illustrates the relationship between various quality parameters of the produce harvested from different fields (Field No. 1 to Field No. 6) and all these fields themselves, revealing significant insights for quality management and physicochemical and biological qualities. Fields No. 1, No. 2, No. 3, and No. 4 cluster closely together, suggesting similar quality attributes, while Fields No. 5 and No. 6 exhibit more distinct profiles, positioned further from the origin along the PC1 and PC2 axes.

The proximity of vectors such as crude protein, crude fat, ash, zinc, and iron to Field No. 5 and Field No. 6 indicates higher concentrations in these fields, contributing to their unique profiles. Parameters like nitrogen, potassium, in vitro starch digestibility, and in vitro protein digestibility are aligned away from the main cluster of fields, indicating lower values in the majority of fields. The biplot underscores the need for tailored quality management practices: enhancing nutrient content in fields with lower levels, addressing specific deficiencies highlighted by the distinct positioning of vectors, and optimizing overall acceptability and sensory attributes such as texture, aroma, and taste based on the unique profiles of each field. By implementing these targeted strategies, the overall quality and productivity of the fields can be improved sustainably and effectively by supplying the nutrition with organic sources and incorporating microbial consortiums.

## 4. Discussion

### 4.1. Response on Soil Nutrients and Quality Parameters

The treatment with organic sources + microbial consortium produced superior values for all the soil physicochemical properties. In the experimental field, the soil was moderately alkaline (pH 8.4) with lower EC (0.13 dS/m); through the increased proportion of chemical fertilizers, the pH value increased continuously and soil saltiness intensified. The gradual increase in organic carbon in organically nourished fields slightly alleviated the soil acidification rate and a minute decrease in the soil pH; that outcome can be explained by the calcium content in organic substances, which act as a buffering agent and keep the soil in the range of acid–base balance. Improved soil nutrient status and soil quality parameters may be due to the higher organic matter, and microbial consortium inoculation could increase the organic carbon content of the soil, which resulted in an enhanced soil water-holding capacity because the humus can absorb water two to six times its weight. The soil organic matter decomposes organic acids, sugars, mucilaginous substances, and gelatinous microbial byproducts that stimulate the formation and stabilization of granular and crumb-type aggregates. The soil microbial consortium can mobilize nutrients from unavailable forms to operational forms through biological processes, which is directly helpful in improving the nutritional quality of the produce. Inoculation of soil microorganisms improves nutrient availability through multifunction biological processes such as acidification of the soil, fixing nitrogen, dissolving potassium, and phosphorus, and also mobilizing micro-nutrients [51]. Plant nutrients are supplied by organic sources continuously, resulting in a more rapid rise in soil organic carbon more and restoring soil fertility and physicochemical properties [52], cumulative available concentrations of nitrogen, phosphorus, potassium, and Fe, Mn, and Zn in sheep manure treatments as compared to chemical fertilizer treatment [53]. The organic sources of crop nourishment were replaced by chemical fertilizers, causing a downward trend in the soil’s availability of N, K, Fe, Zn, and Cu [54,55]. Excessive chemical fertilization delivers nutritious elements for soil, resulting in a deficiency of organic matter; it may lead to soil degradation, reduce organic carbon, and start incessant fertility decline [2,56], and significant loss of soil biodiversity. Because of the above facts and the constant negative effects on vegetable productivity and nutritional quality, this has led to auspicious demand from society for reduced use of chemical fertilizers in agricultural production [57].

### 4.2. Response to Soil Biological Population

The organic nutrition with microbial vaccination considerably boosted soil biological activities such as population density of soil fungi, bacteria, Trichoderma, actinomycete, and earthworm. In contrast, the intensive use of chemical fertilizers regularly contributes to a loss in the microbial and earthworm count significantly. A possible cause behind the multiplication in the microbes is that organic substances significantly affect the promotion of microbial biomass, mainly because soil organic carbon is the limiting factor for microbial reproduction, and the addition of microbial consortium increases the amount of carbon input into the system. Similarly, adding organic manure to the soil led to a significant intensification in the soil microbial population and biomass [53]. Research has also demonstrated that the collective application of organic manure and microbial consortium significantly enhances the metabolic activities of beneficial microorganisms and earthworms in the experimental field; this was also proved by Yao et al. [58] and Wang et al. [59]. Organic sources of nutrition mitigate soil degradation and remedy nutrient loss to improve soil organic carbon storage [60], and they may also enhance soil microbial biomass and change microbial community structure and soil health [61,62]. Researchers previously discovered that the long-term application of organic substances could alter the spatial scaling of microbial biodiversity [63] and stimulate microbiota such as firmicutes, proteobacteria, and Zygomycota [64]. Application of green manure considerably increases the copiousness of *Chryseolinea* [65], *Bacillales*, and *Gaiellales* [66], whereas sheep manure resulted in a proliferation of gamma proteobacteria, *Chlamydiae*, *Bastocatellia*, and *Clostridia* in the soil rhizosphere [2]. Oppositely, the long-term chemical fertilizer application decreases soil microorganisms and soil microbial biomass, especially *Actinobacteria* [53], *Pseudodoganella* [65], and decreases the complexity of networks between plant and microbial functional communities [67]. Reasonable uses of inorganic and organic amendments with microbial inoculation can effectively increase soil nutrient content, improve soil physical and chemical conditions, and stimulate the metabolic intensity of soil microorganisms, which is essential for agricultural production sustainability.

### 4.3. Response of Plant Growth and Yield Attributes

**Figure 3:**
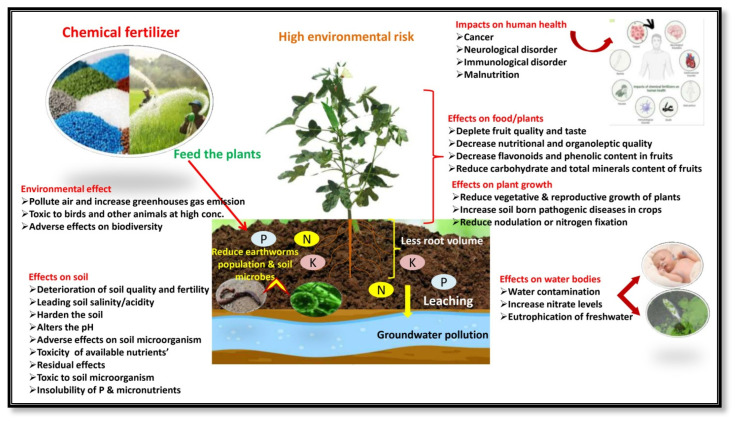
Impacts of chemical fertilizers on the environment and food quality of vegetable crops.

**Figure 4:**
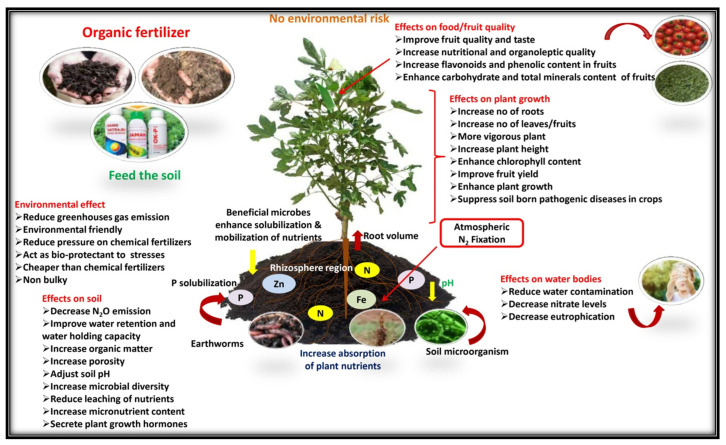
Organic fertilizers (biofertilizer; arbuscular mycorrhiza fungi; plant growth-promoting rhizobacteria) affect plant production of health-promoting phytochemicals of vegetable crops and the surrounding environment.

The experimental results of plant growth and fruit yield showed that the nutrients supplied by organic sources with microbial inoculation treatment (Field No. 6) were found to be superior to other treatments due to favorable soil physical conditions, enhanced nutrient availability, and absorbability to plants. The treatments had significant effects on growth and yield attributes, with an increasing proportion of organic sources of nutrition up to 100% (Field No. 5), and the fruit yield and net income slightly decreased compared with chemical sources alone (Field No. 1). It may be explained by the fact that the decomposition rate of mineral nutrients in chemical fertilizer is faster than that of organic sources, making it easier and quicker to keep up with the rapid growth of plants. The release of nitrogen and other nutrients sufficiently from organic substances in the presence of microbial consortium (Field No. 6) throughout the growing period might have contributed to the considerable vegetative growth and increase in fruit yield of tomato (18.32%), brinjal (13.58%), and okra (16.23%) when compared to integrated as well as chemical source treatment. As a consequence of the continuous increase in the use of chemical fertilizer and the corresponding decrease in organic manure practice, the number and diversity of soil microbial flora and fauna reduced, and the reduction in soil fertility led to the gradual decline in vegetable productivity (Figure 3), whereas microbial inoculation stimulates decomposition, fixation, and nutrient solubility, resulting in increased nutrient availability to plants and enhanced productivity of the growing crops (Figure 4). The supporting effect of PGPMs may be that microorganisms increase the root activity in the rhizosphere, initiate hormonal action, and thus increase the uptake of plant nutrients. The results were similar to the findings of Song et al. [68] and Zhai et al. [69], who reported that integrating a 10%–20% reduction in chemical fertilizer usage with the appropriate application of bio-organic fertilizer and mixed organic and inorganic fertilizer [2] can enhance soil fertility, promote the growth and development of plants, and ultimately increase productivity. The hypothesis behind the above facts is that the combined application of microbial consortium can activate and balance soil nutrient system, and enhance crop absorption and utilization, which helps in increasing leaf nutrient content, thereby effectively improving photosynthetic efficiency, and this is beneficial for the accumulation of plant biomass, minimizing redundant growth, and promoting a more efficient distribution of photosynthetic products to reproductive organs [18,70]. A similar response of the microbial consortium inoculation was also observed by Miskoska-Milevska et al. [71] in cauliflower, Seif Sahandi et al. [72] in mint, Zhao et al. [54] in cucumber and Sousa et al. [73] and Demir et al. [74] in lettuce.

### 4.4. Response on Nutritional and Organoleptic Qualities of Fruits

The principal causes of the nutrient decline are the degradation of the soil qualities, trimming down soil microbial population with a radical reduction in soil bio-diversity, and severe disturbance in the soil ecosystem [6] due to the long-term excessive or imbalanced use of synthetic fertilizers (Figure 3). The fruit moisture content (89.82%) was the lowest and crude fat (0.49%), ash (0.88%), fiber (3.56%), total sugars (3.64%), total mineral content, overall acceptability, in vitro protein, and starch digestibility were significantly higher than those of other treatments with a certain proportion of chemical fertilizer without microbial consortium. The best performance in improving proximate composition, available carbohydrate, total mineral content, and organoleptic quality with significantly enhanced protein and starch digestibility was reported with the microbial consortium inoculation (PGPM) treatments (Figure 4).

The quality analysis results of tomato, brinjal, and okra fruits indicate that the combined application of chemical fertilizer and organic manure also enhances the contents of minerals and organoleptic qualities while reducing the moisture content of fruits. A conceivable reason behind the perfection in the nutritional and organoleptic qualities of fruits is that microbial consortium (PGPM) can convert nutritionally important elements from unavailable forms to available forms through biological processes [75]. Similar results were also reported by many researchers, such as biofertilizer application increasing lettuce nutrient (K, Mg, Na, P, Fe, Zn, and N) concentration [76], PGPR inoculation with *Pseudomonas fluorescens* for lettuce plants significantly increasing the shoot dry matter and N, P, Ca, Mg, Mn, and Na uptake rates [73,77] and also increasing the sweetness, secondary metabolites, antioxidant properties, and content of minerals, as well as chlorophylls in vegetables [78], and Azotobacter and phosphate solubilizing bacteria treatments increasing the zinc content in broccoli [79]. Field soil enriched with bacteria and rhizospheric microorganisms (PGPMs) resulting in increases in the availability and absorbability of microelements by plants, which are helpful in the synthesis of various phytochemicals; these encourage organoleptic quality and also recover the vitamin, flavonoid, antioxidant, and mineral contents [80]. These results play an important role in guiding fertilization strategies for maintaining the nutritional and organoleptic qualities of vegetables and provide a research basis for the synergistic use of chemical fertilizers, organic manure, and microbial consortium for tomato, brinjal, and okra cultivation.

### 4.5. Response on Nutraceutical and Antioxidant Quality of Fruits

The present study showed that in the fruits of tomato, brinjal, and okra nutrients, total phenolic, flavonoids, soluble sugars, and overall acceptability upsurged to varying degrees in the experimental Field No. 1 to Field No. 6. The possible cause behind enhanced nutraceutical and antioxidant quality of fruits under the organic production system with microbial inoculation (PGPM) is that the unique microbial community can activate bio-stimulants in the field soil, improve the physico-chemical properties, increase soil biodiversity, and enhance soil enzyme activity [81]. All these factors effectively extend the plant root system with mycelium, releasing nutrients from the soil for plants and enhancing diversified nutrients and stimulants absorbed by the plant root system, which are responsible for the synthesis of more phytochemicals, such as antioxidants, flavonoids, and phenolic compounds in fruits. The phenolic compounds were found to be 20 percent higher in organic products as compared to conventional products [82]. Similarly, 35 to 60 percent more antioxidants, vitamins, and significant levels of ascorbic acid and lycopene were found under the organic system with mycorrhizal inoculation in different fruits and vegetables [83], in lettuce [73,84] and edible roses [70]. Apposite practices of organic substances in combination with bio-organic and chemical fertilizers enhance the nutraceutical quality of crops [85] and significantly increase the nutritional content of purslane, including total flavonoids and phenolic compounds, while reducing anti-nutritional compounds such as nitrates and soluble oxalic acid [86], which helps in increasing the digestibility of the produce.

## 5. Conclusions

The positive responses were found in the nutrition supplied by the organic sources in the presence of PGPMs for tomato, brinjal, and okra concerning improving the physico-chemical and biological qualities of field soil, nutritional, organoleptic, and nutraceutical quality of fruits. The outcomes are extremely remarkable because of the continuing increase in the price of chemical fertilizers used in vegetable farming and their long-term detrimental consequences on the environment, soil, and human health. In conclusion, the PGPMs had supportive effects on the growth parameters, yield, quality, and mineral concentrations in tomato, brinjal, and okra fruits. Moreover, when PGPMs are used, it should be considered that the positive effects will continue to increase on the beneficial microorganism activity and earthworm counts in the cultivable soil, and as a result, provide long-term sustainability with positive effects on human health, soil quality, biodiversity, and the stability of ecosystems. Consequently, future investigations should primarily focus on the influence of the combination of organic substances with the microbial consortium for the gradual reduction in chemical fertilizers for vegetable production to further elucidate the underlying mechanisms governing their association with both yield and quality.

## Figures and Tables

**Table 1:** Initial (2019–2020) physicochemical properties of the experimental field soil.

Particulars	Status	References
A. Physical Properties		
Sand (%)	62.0	[20]
Silt (%)	30.0	
Clay (%)	8.0	
Texture class	Sandy loam	[21]
Bulk density (g cm^−3^)	1.43	[22]
Particle density (g cm^−3^)	2.39	
Total porosity (%)	39.92	
Infiltration rate (mm/h)	3.13	
Water holding capacity (%)	25.44	
Moisture (%)	11.29	
B. Chemical Properties		
pH (1:2.5 soil water suspension)	8.40	[23]
Electrical conductivity (dS/m)	0.13	[23]
Organic carbon (%)	0.29	[24]
KMnO_4_ oxidizable N (kg/ha)	220.0	[25]
0.5 M NaHCO_3_-extractable P (kg/ha)	12.52	[26]
Neutral 1 N NH_4_OAc-extractable K (kg/ha)	225.4	[23]

**Table 2:** Effects of nutrition supply sources on soil fertility, physico-chemical quality, and soil biological activities of the vegetable production field soil during experimentation.

Quality Parameters	Initial Values (2019–2020)	Tomato, Brinjal, and Okra Growing Fields in Arid Climate (2023–2024)								
		Field No. 1	Field No. 2	Field No. 3	Field No. 4	Field No. 5	Field No. 6	S. Em +	C.D. (*p* = 0.05)	% Change
Nutritional status of the upper layer of soil (0–30 cm)										
O.C. (%)	0.29	0.18	0.34	0.42	0.42	0.44	0.70	0.006	**	141.38
N_2_ (Kg/ha)	220.0	237.50	210.40	203.4	198.7	165.7	225.0	6.124	**	2.27
P_2_O_5_ (Kg/ha)	37.5	26.50	31.00	35.00	49.00	54.00	72.50	0.612	**	93.33
K_2_O (Kg/ha)	225.4	206.5	224.0	252.0	275.8	292.5	312.0	6.531	**	38.42
S (ppm)	7.55	8.62	8.11	7.58	6.92	6.25	7.11	0.245	**	−5.83
Zn (ppm)	0.35	0.23	0.38	0.54	0.65	0.78	1.18	0.010	**	237.14
Fe (ppm)	1.85	1.36	1.85	2.00	2.97	3.08	3.37	0.037	**	82.16
Cu (ppm)	0.60	0.46	0.55	0.88	0.90	1.02	1.12	0.012	**	86.87
Mn (ppm)	5.99	6.40	6.64	6.96	7.15	7.80	8.48	0.049	**	41.57
Physico-chemical quantity of the upper layer of soil (0–30 cm)										
EC	0.13	0.11	0.15	0.19	0.22	0.32	0.47	0.015	**	261.54
pH	8.40	8.50	8.20	8.20	8.00	7.80	7.60	0.057	**	−9.52
Bulk density	1.43	1.45	1.46	1.42	1.42	1.32	1.30	0.029	**	−9.09
Particle density	2.39	2.30	2.33	2.40	2.44	2.30	2.27	0.037	**	−5.02
Porosity (%)	39.92	36.96	37.34	40.83	41.80	42.17	42.73	0.453	**	7.04
Infiltration Rate (mm/hr)	3.13	3.01	3.18	4.02	5.21	6.12	6.52	0.045	**	108.31
MWHC (%)	25.44	25.05	26.30	29.34	33.96	37.54	37.62	0.461	**	47.88
Moisture (%) #	11.29	10.99	11.12	13.58	14.21	14.98	15.11	0.349	**	33.84
Water saving (%)	00.0	−1.56	3.27	13.29	25.09	32.23	32.38	0.327	**	32.38
Soil biological population in the upper layer of soil (0–30 cm)										
Fungi (CFU)	3 × 10^−3^	0.00	0.00	0.00	5 × 10^−3^	25 × 10^−3^	92 × 10^−3^	0.230	**	2966.7
Bacteria (CFU)	6 × 10^−3^	0.00	0.00	0.00	8 ×10^−3^	22 × 10^−3^	82 × 10^−3^	0.322	**	1267.0
Trichoderma (CFU)	1 × 10^−3^	0.00	0.00	0.00	3 × 10^−3^	19 × 10^−3^	97 ×10^−3^	0.610	**	9600.0
Actinomycete (CFU)	4 × 10^−3^	0.00	0.00	3 × 10^−3^	5 × 10^−3^	25 × 10^−3^	75 × 10^−3^	0.491	**	1775.0
Earthworm ##	20.0	0.00	0.00	3.50	15.83	27.50	29.83	0.429	**	49.15

Field No. 1 = 100% Chemical source of nutrients; Field No. 2 = 75% Chemical source + 25% Organic source; Field No. 3= 50% Chemical source + 50% Organic source; Field No. 4 = 25% Chemical source + 75% Organic source; Field No. 5= 100% Organic source; Field No. 6 = 100% Organic source + Microbes (PGPMs) inoculation; ** 5% level of significance (*p* = 0.05); ## Earthworm population (no. of worm square m^−1^); NS—Non-significant; MHWC—mean water holding capacity; # moisture content (%) at field capacity.

**Table 3:** Effects of nutrient supply sources on vegetative growth and yield attributes of vegetables.

Quality Parameters	Field No. 1	Field No. 2	Field No. 3	Field No. 4	Field No. 5	Field No. 6	S. Em +	C.D. (*p =* 0.05)	Percent Change
Tomato fruits									
Plant height (cm)	101.11	97.51	95.03	87.84	79.38	104.91	1.461	**	3.76
Number of leaves	215.45	193.22	191.78	178.15	165.45	236.74	1.869	**	9.88
Root length (cm)	13.25	11.23	10.89	10.61	9.51	14.65	0.237	**	10.57
No. of roots/plant	18.97	19.11	20.66	21.45	22.89	24.15	0.318	**	27.31
No. of fruits/plants	37.23	35.44	32.14	28.79	27.55	42.35	0.400	**	13.75
Fruit weight (g)	88.75	85.56	82.54	80.11	79.56	99.15	1.217	**	11.72
Fruit yield (tons/ha)	42.15	40.15	39.87	37.81	35.11	49.87	0.808	**	18.32
Total income (INR L/ha)	2.56	2.50	2.31	2.19	2.14	2.97	0.033	**	16.02
Net income (INR L/ha)	1.90	1.82	1.58	1.43	1.34	2.05	0.037	**	7.89
B:C Ratio	2.93	2.68	2.20	1.91	1.68	2.23	0.045	**	−23.89
Brinjal fruits									
Plant height (cm)	68.23	67.99	65.14	60.15	58.25	72.58	0.673	**	6.38
Number of leaves	101.15	95.82	89.56	82.44	72.15	109.72	1.270	**	8.47
Root length (cm)	14.32	13.65	13.22	12.65	11.15	14.98	0.610	**	4.61
No. of roots/plant	10.14	10.22	10.27	11.24	13.25	14.58	0.078	**	43.79
No. of fruits/plants	29.58	27.12	25.46	24.01	20.17	32.55	0.486	**	10.04
Fruit weight (g)	78.52	75.44	72.15	70.89	65.48	85.47	0.510	**	8.85
Fruit yield (tons/ha)	37.48	35.51	32.14	30.55	27.12	42.57	0.645	**	13.58
Total income (INR L/ha)	2.44	2.31	2.09	1.99	1.76	2.77	0.033	**	13.52
Net income (INR L/ha)	1.77	1.61	1.35	1.22	0.94	1.85	0.029	**	4.52
B:C Ratio	2.64	2.30	1.82	1.58	1.15	2.01	0.037	**	−23.86
Okra fruits									
Plant height (cm)	94.26	91.22	89.65	88.50	85.14	106.41	0.853	**	12.89
Number of leaves	31.54	28.56	25.01	21.14	17.66	37.45	0.445	**	18.74
Root length (cm)	18.20	16.11	14.60	14.27	13.51	21.77	0.363	**	19.62
No. of roots/plant	24.07	25.56	28.14	32.15	35.46	37.28	0.404	**	54.88
No. of fruits/plants	12.45	11.33	10.87	10.13	9.56	14.56	0.326	**	16.95
Fruit weight (g)	8.58	7.61	8.31	7.89	7.56	9.25	0.204	**	7.81
Fruit yield (tons/ha)	10.23	10.01	9.23	8.74	8.56	11.89	0.184	**	16.23
Total income (INR L/ha)	2.56	2.51	2.31	2.19	2.14	2.97	0.021	**	16.02
Net income (INR L/ha)	1.92	1.83	1.58	1.43	1.34	2.05	0.022	**	6.77
B:C Ratio	2.93	2.68	2.21	1.91	1.68	2.23	0.028	**	−23.89

Field No. 1 = 100% Chemical source of nutrients; Field No. 2 = 75% Chemical source + 25% Organic source; Field No. 3 = 50% Chemical source + 50% Organic source; Field No. 4 = 25% Chemical source + 75% Organic source; Field No. 5 = 100% Organic source; Field No. 6 = 100% Organic source + Microbe (PGPM) inoculation., ** 5% level of significance (*p* = 0.05).

**Table 4:** Effects of nutrient supply sources on nutritional and organoleptic quality of vegetables grown in arid climate conditions.

Quality Parameters	Tomato, Brinjal, and Okra (100 g of Vegetable Pulp)								
	Field No. 1	Field No. 2	Field No. 3	Field No. 4	Field No. 5	Field No. 6	S. Em +	C.D. (*p* = 0.05)	% Change
Proximate composition									
Moisture (%)	92.04	91.37	91.08	90.30	89.96	89.82	2.368	NS	−2.41
Crude protein (%)	2.10	2.08	2.01	1.91	1.91	1.96	0.033	**	−6.67
Crude fat (%)	0.34	0.35	0.38	0.42	0.45	0.49	0.003	**	44.12
Ash (%)	0.65	0.68	0.70	0.75	0.80	0.88	0.020	**	35.38
Crude fiber (%)	3.22	3.17	3.21	3.38	3.42	3.56	0.038	**	10.56
Total carbohydrates (g)	85.07	85.23	85.33	85.22	85.29	85.43	2.041	NS	0.42
Available carbohydrates									
Total sugars (%)	1.11	1.31	1.65	1.79	3.29	3.64	0.020	**	227.93
Reducing sugar (%)	0.59	0.80	0.96	1.03	2.37	2.59	0.032	**	338.98
Non-reducing sugars (%)	0.52	0.51	0.69	0.76	0.92	1.05	0.012	**	101.92
Total minerals									
Calcium (mg)	37.49	37.83	38.12	39.16	39.66	39.74	0.400	**	6.00
Phosphorus (mg)	36.13	36.42	36.67	37.45	38.29	38.47	0.416	**	6.48
Iron (mg)	0.82	0.88	0.93	1.01	1.08	1.20	0.008	**	46.34
Zinc (mg)	0.27	0.29	0.32	0.36	0.41	0.52	0.004	**	92.59
Magnesium (mg)	33.69	34.14	34.29	34.56	35.43	38.80	0.772	**	15.17
Nitrogen (g)	0.71	0.69	0.69	0.68	0.72	0.76	0.006	**	7.04
Potassium (mg)	237.20	237.39	238.01	240.26	241.41	244.37	2.864	**	12.14
Organoleptic evaluation of fresh vegetables by the 9-point hedonic mean scale									
Appearance	7.34	7.40	7.39	7.48	7.58	7.81	0.082	**	6.40
Color	7.71	7.78	7.82	7.96	8.23	8.37	0.118	**	8.56
Aroma	6.97	7.05	7.30	7.53	7.69	7.91	0.143	**	18.65
Taste	6.42	6.55	7.02	7.59	7.88	8.12	0.184	**	32.87
Texture	7.49	7.55	7.63	7.79	8.00	8.22	0.163	**	12.42
Overall acceptability	7.18	7.27	7.43	7.68	7.88	8.30	0.157	**	15.60
Digestibility									
In vitro protein Digestibility (%)	74.48	75.34	77.12	78.09	79.54	78.04	1.020	**	4.78
In vitro starch Digestibility (%)	63.76	65.99	67.81	67.85	69.10	70.99	1.053	**	11.34

Field No. 1 = 100% Chemical source of nutrients; Field No. 2 = 75% Chemical source + 25% Organic source; Field No. 3 = 50% Chemical source + 50% Organic source; Field No. 4 = 25% Chemical source + 75% Organic source; Field No. 5 = 100% Organic source; Field No. 6 = 100% Organic source + Microbe (PGPM) inoculation; NS—Non-significant; ** 5% level of significance (*p* = 0.05).

**Table 5:** Effects of nutrient supply sources on nutraceutical quality and antioxidant content in fruit vegetables grown in arid climate conditions.

Quality Parameters	Field No. 1	Field No. 2	Field No. 3	Field No. 4	Field No. 5	Field No. 6	S. Em +	C.D. (*p* = 0.05)	% Change
Tomato fruits									
Total flavonoids (mg QE/g)	2.21	2.26	2.35	2.47	2.63	3.04	0.073	**	37.56
Total phenolic content (mg GAE/g)	3.51	5.21	6.45	7.46	7.92	8.74	0.068	**	41.14
Radicals Scavenging Activity (DPPH, µmol Trolox/g)	10.27	11.29	11.49	12.64	13.87	14.20	0.351	**	38.27
^#^ FRAP (µmol Trolox/g) assay	14.73	15.21	16.09	17.66	18.49	19.27	0.340	**	30.82
^##^ ABTS (µmol Trolox/g) assay	28.20	29.48	31.08	32.88	33.66	34.11	0.745	**	20.96
Brinjal fruits									
Total flavonoids (mg QE/g)	4.67	4.73	4.96	5.19	5.60	6.34	0.113	**	35.84
Total phenolic content (mg GAE/g)	5.49	6.25	7.20	7.61	8.46	8.84	0.153	**	42.81
Radicals Scavenging Activity (DPPH, µmol Trolox/g)	4.71	4.82	4.97	5.27	6.24	7.14	0.133	**	51.60
^#^ FRAP (µmol Trolox/g) assay	4.61	4.74	4.95	5.18	5.69	6.84	0.129	**	48.37
^##^ ABTS (µmol Trolox/g) assay	10.11	10.98	12.01	12.91	13.40	14.12	0.365	**	39.66
Okra fruits									
Total flavonoids (mg QE/g)	2.48	2.52	2.63	2.81	2.97	3.62	0.014	**	45.97
Total phenolic content (mg GAE/g)	2.79	2.85	2.97	3.12	3.65	4.15	0.055	**	48.75
Radicals Scavenging Activity (DPPH, µmol Trolox/g)	4.23	4.88	5.15	5.85	6.31	6.52	0.076	**	54.14
# FRAP (µmol Trolox/g) assay	6.15	6.48	6.78	7.12	8.14	8.54	0.089	**	36.86
## ABTS (µmol Trolox/g) assay	19.67	21.66	22.40	23.22	24.02	24.85	0.496	**	26.33

Field No. 1 = 100% Chemical source of nutrients; Field No. 2 = 75% Chemical source + 25% Organic source; Field No. 3 = 50% Chemical source + 50% Organic source; Field No. 4 = 25% Chemical source + 75% Organic source; Field No. 5 = 100% Organic source; Field No. 6 = 100% Organic source + Microbes (PGPMs) inoculation; ** 5% level of significance (*p* = 0.05); # Ferric Reducing Ability of Plasma; ## 2,2′-azino-bis-3-ethylbenzothiazoline-6-sulfonic acid.

## Data Availability

The original contributions presented in the study are included in the article, further inquiries can be directed to the corresponding author.
